# Plugging the Nasal Cavities

**Published:** 1879-08

**Authors:** 


PLUGGING THE NASAL CAVITIES.—Roll up a lock of cotton into a cylinder an inch or an inch and a half in length; tie a strong thread to the middle of the roll; bring the two ends of the roll together, and then, opening the nasal orifice, pass the middle or folded part of the roll into the nostril; next, with the blunt end of a lead pencil, press in the cotton roll slowly along the floor of the nose an inch or more, and rest. If the blood passes down into the throat, you may be sure the bleeding spot is behind the roll; so push in your roll further and the blood will cease to pass behind. Then, holding on to the string, pass some loose cotton in the nostril and push it down to the plug. The cotton will swell with the moisture and arrest the hemorrhage. In a day or two the natural secretions of the nasal surfaces will loosen the plug, and it may be easily removed by the string.—*Southern Clinic.*

